# Increased Risk of Suicide Attempts Among Adolescents With Food Allergy in the United States

**DOI:** 10.1016/j.jaacop.2024.11.005

**Published:** 2024-11-29

**Authors:** Saurabh Kalra, Evan M. Kleiman, Shireen L. Rizvi, Irina B. Grafova, Paul R. Duberstein, Deepak Kalra

**Affiliations:** a Miller School of Medicine, University of Miami, Miami, Florida; b Rutgers University, New Brunswick, New Jersey; c Penn State College of Medicine, Pennsylvania State University, Hershey, Pennsylvania

**Keywords:** adolescent, food allergy, food hypersensitivity, mental health, suicide

## Abstract

**Objective:**

Food allergy (FA) is a growing public health concern that has been associated with feelings of sadness, hopelessness, shorter sleep duration, and being bullied—all linked to suicide risk. Given the limited prior research, this study aimed to examine the association between FA and suicidal behavior.

**Method:**

Data from the Centers for Disease Control and Prevention Youth Risk Behavior Survey (2015-2019) were analyzed. This nationally representative sample included 22,017 adolescents 14 to 18 years old. To assess the association between FA and medically serious suicide attempts, several multivariate logistic regressions were conducted adjusting for effects of sadness/hopelessness, suicidal ideation, shorter sleep duration, being bullied, physical activity, and other covariates. Additionally, a sensitivity analysis was conducted to explore the association between FA and all suicide attempts, including attempts not requiring medical attention.

**Results:**

Among the participants, 15.6% (n = 3,435) reported FA, and 2.3% (n = 501) reported serious suicide attempts. Within the serious suicide attempt group, 31.9% (n = 160) reported FA compared with 15.2% (n = 3,275) among participants who did not report serious suicide attempts. Multivariate analyses revealed that adolescents with FA had higher odds of reporting suicide attempts requiring medical intervention (adjusted odds ratio 1.63, 95% CI 1.23-2.16). In the sensitivity analysis, FA continued to show significance when broadening the outcome to include all suicide attempts, regardless of whether medical attention was required (adjusted odds ratio 1.35, 95% CI 1.09-1.68).

**Conclusion:**

Health care practitioners caring for adolescents with FA should be cognizant of their increased susceptibility for suicidal behavior.

## Plain language summary

Adolescents with food allergies have a higher risk of serious suicide attempts. The study found that those with food allergies were 63% more likely to attempt suicide compared to their peers without food allergies. Clinicians should consider mental health risks in adolescents with food allergies and offer appropriate support.

Food allergy (FA) is increasingly recognized as a significant public health issue in adolescents, affecting approximately 10% of adolescents in the United States.^1^^,^^2^ With FA being linked to common risk factors for suicide,^3^, ^4^, ^5^ such as health behaviors (eg, sleep disturbances)^6^, ^7^, ^8^ and feelings of sadness and hopelessness,^9^, ^10^, ^11^ there is possibility of an association between FA and suicidal behavior. However, despite this potential link, prior research has not thoroughly explored the association between A and suicide attempts.

Numerous studies have shown that FA in adolescents is associated with heightened levels of anxiety, sadness, depression, sleep disturbances, and feelings of hopelessness.^10^^,^^11^ For instance, in the Great Smoky Mountains longitudinal cohort study including 1,492 adolescents recruited from 11 counties in North Carolina, FA was found to predict an escalation in depressive symptoms from ages of 10 to 16.^11^ Furthermore, FA has been associated with incidents of being bullied.^12^^,^^13^^,^^14^ Research from Italy comprising 120 adolescents with FA and an equal number without FA revealed that adolescents with FA were 1.89 times more likely to experience bullying compared with their healthy counterparts.^13^ Additionally, there is evidence of an association between shorter sleep duration and FA.^7^^,^^8^ A study including 1,534 adolescents from China showed that FA was nearly twice as prevalent among participants sleeping less than 8 hours per night.^8^ Despite the well-established connections between feelings of sadness, hopelessness, bullying, shorter sleep duration, and suicide risk,^3^, ^4^, ^5^^,^^15^, ^16^, ^17^ investigations into the association between FA and suicide remain scarce. This absence is unexpected given the existence of a theoretical framework proposing a link between allergies and suicidal behavior, positing that allergies induce neuroinflammation, which in turn may be associated with suicidal tendencies.^18^

Using data from the 2015 through 2019 Youth Risk Behavior Surveys (YRBS),^19^ we aimed to explore the association between FA and serious suicide attempts, defined as suicide attempts resulting in injuries necessitating medical attention. The YRBS, administered by the US Centers for Disease Control and Prevention (CDC) since 1990, is a crucial tool for assessing the health risk behaviors of school students.^19^^,^^20^ Administered by the CDC, states, and local district health and education offices, the Youth Risk Behavior Surveillance System database is instrumental in advancing evidence-based policymaking and research efforts dedicated to enhancing adolescent well-being.^21^ The YRBS offers comprehensive coverage of various covariates, making it particularly well suited for our investigation.

We sought to determine whether the association between FA and serious suicide attempts persists independently of established correlates of suicide attempts, such as feelings of sadness/hopelessness, shorter sleep duration, and experiences of bullying.^3^, ^4^, ^5^^,^^15^ We hypothesized that even after adjusting for these covariates, FA would demonstrate an independent association with suicide attempts.

## Method

### Data Source

**Figure 1:**
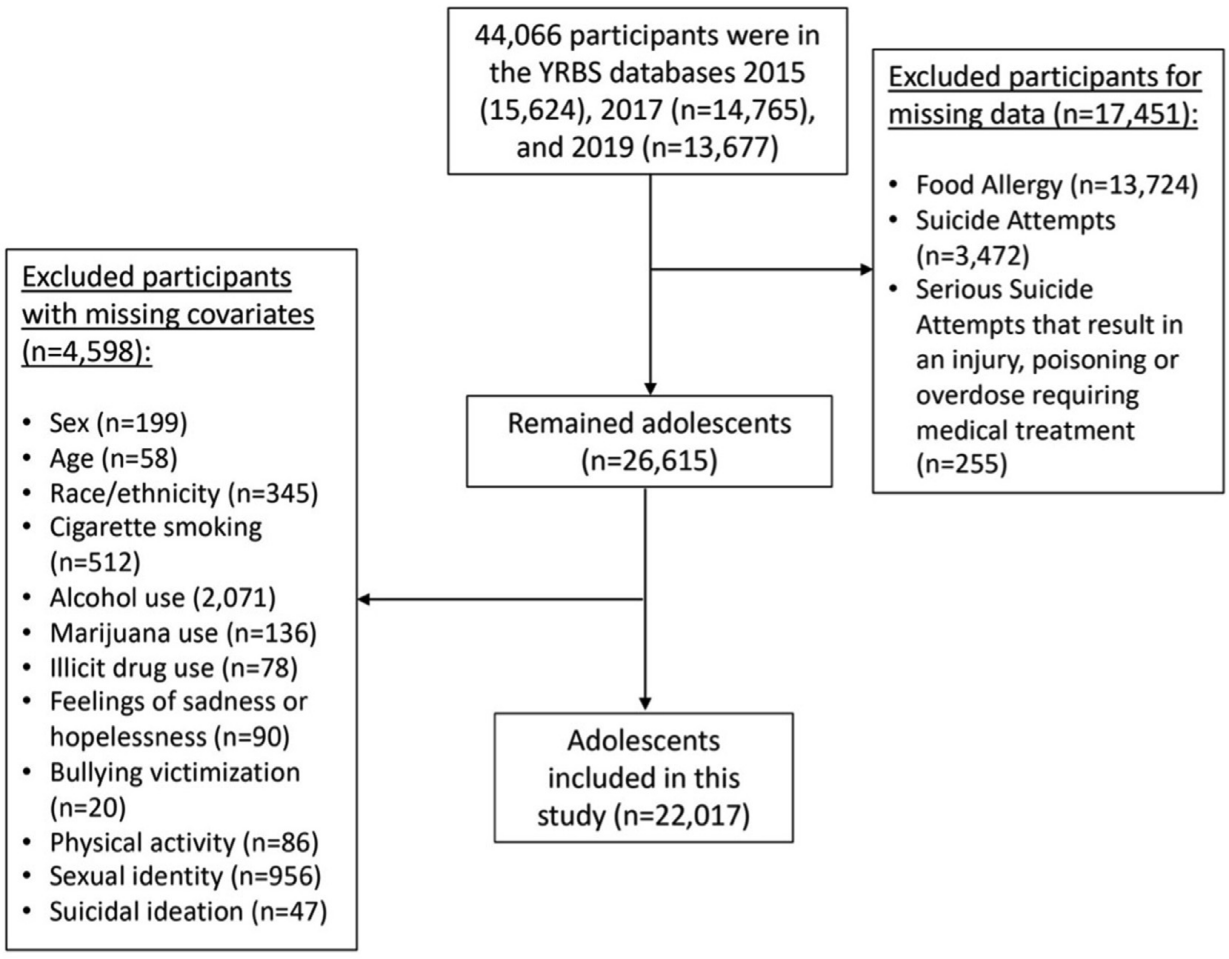
Flowchart for Study Population Selection, Youth Risk Behavior Survey (YRBS) Databases 2015-2019

The YRBS is conducted biennially and is an anonymous, voluntary survey employing a 3-stage cluster sampling technique to obtain a nationally representative sample of US adolescents in grades 9 through 12 from both public and private schools across the 50 US states and the District of Columbia. We combined data from 3 YRBS administrations (2015: n = 15,624; 2017: n = 14,765; 2019: n = 13,677) as questions regarding FA were not included before 2015 or after 2019. Among the 44,066 respondents, 26,870 provided responses to both FA and suicide attempt questions. Our final analytic sample was 22,017 after excluding participants who did not respond to at least 1 item about suicide attempts (n = 255), demographics (n = 1,558), bullying (n = 20), sadness or hopelessness (n = 90), suicidal ideation (n = 47), and health behaviors (n = 2,883) (Figure 1). The most frequently omitted item among health behaviors was regarding alcohol intake (n = 2,071). The overall weighted survey response rate ranged from 59.3% to 60.3%.^20^^,^^22^ Further information regarding the sampling methodology and survey procedures can be accessed in the publicly available data release.^20^^,^^22^

### Measures

#### Dependent Variable: Serious Suicide Attempt

Survey participants were asked: “During the past 12 months, if you attempted suicide, did any attempt result in an injury, poisoning, or overdose requiring treatment by a doctor or nurse?” Responses were documented as either “yes” or “no.”

#### Independent Variable: FA

Survey respondents were asked about whether there are any foods that they need to avoid because consuming them could trigger an allergic reaction, such as skin rashes, swelling, itching, vomiting, coughing, or difficulty breathing. Responses were documented as either “yes” or “no.”

#### Covariates

The multivariable model was adjusted for the following variables: sex; race/ethnicity; sexual identity; receiving less than 6 hours of sleep on an average school night; past-month use of cigarettes, alcohol, or marijuana; lifetime use of specific illicit drugs, including cocaine, inhalants, heroin, methamphetamines, ecstasy, or hallucinogens; feelings of hopelessness or sadness within the past year; experiencing serious suicidal ideation within the past year; being bullied on school property or electronically (via texting, Facebook, or Instagram) within the past year; engaging in physical activity for more than 60 minutes on at least 5 days in the last week; and survey years.

### Statistical Analysis

Descriptive statistics were conducted to provide a comprehensive overview of the data based on suicide attempts requiring medical attention (Table 1) and FA status (Table 2). The χ^2^ test was used to explore the bivariate associations. Based on the test statistics, the variables deemed pertinent for predicting suicide attempts were included in the multivariate logistic regression models to study associations between FA and serious suicide attempts. We also evaluated for any demographic differences between the participants included in the study and youth excluded from the study due to missing data. These comparisons are detailed in Tables S1 and S2 (available online).

**Table 1:** Descriptive Statistics of 22,017 High School Adolescents Based on Serious Suicide Attempt Requiring Medical Treatment

Variables	Serious suicide attempt^a^ (n = 501 [2.3%])		Remaining respondents (n = 21,516 [97.7%])		*p*^b^
	n	(%)	n	(%)	
Sex					<.001
Female	385	(76.9)	11,129	(51.7)	
Male	116	(23.2)	10,387	(48.3)	
Age, y					.58
14	51	(10.2)	2,290	(10.6)	
15	133	(26.6)	5,222	(24.3)	
16	131	(26.2)	5,609	(26.1)	
17	128	(25.6)	5,421	(25.2)	
≥18	58	(11.6)	2,974	(13.8)	
Race/ethnicity					<.001
Hispanic/Latinos	64	(12.8)	2,414	(11.2)	
Non-Hispanic African American	60	(12.0)	2,264	(10.5)	
Non-Hispanic others	64	(12.8)	2,414	(11.2)	
Non-Hispanic White	212	(42.3)	10,787	(50.1)	
Alcohol use, past month	288	(57.5)	6,514	(30.3)	<.001
Cigarette smoking, past month	147	(29.3)	1,652	(7.7)	<.001
Marijuana use, past month	254	(50.7)	4,190	(19.5)	<.001
Illicit drug use, ever^c^	208	(41.5)	2,450	(11.4)	<.001
Sleeping <6 h on an average school night	207	(41.3)	4,291	(19.9)	<.001
Bullied in school, past year	279	(55.7)	3,960	(18.4)	<.001
Bullied electronically through texting or social media, past year	252	(50.3)	3,039	(14.1)	<.001
Physically active >60 min/day on ≥5 days, past week	204	(40.7)	10,191	(47.4)	<.001
Felt sad or hopeless, past year	472	(94.2)	6,901	(32.1)	<.001
Suicidal ideation, past year	484	(96.6)	3,568	(16.6)	<.001
Suicide attempt, past year	1,190	(5.5)	500	(100)	
Year					.147
2015	207	(41.3)	8,014	(37.3)	
2017	158	(31.5)	7,427	(34.5)	
2019	136	(27.2)	6,075	(28.2)	
FA^d^	160	(31.9)	3,275	(15.2)	<.001

Note: Youth Risk Behavior Survey (YRBS) databases 2015-2019. FA = food allergy.

a Respondents were asked if the suicide attempt resulted in an injury, poisoning, or overdose that required medical attention.

b The χ^2^ test was used for independence. All analyses used YRBS survey weights.

c Respondents were asked if they ever used specific illicit drugs, including cocaine, inhalants, heroin, ecstasy, methamphetamines, or hallucinogens.

d Respondents were asked if there are any foods that they must avoid because eating the food could cause an allergic reaction, such as skin rashes, swelling, itching, vomiting, coughing, or trouble breathing.

**Table 2:** Descriptive Statistics of 22,017 High School Adolescents Based on Food Allergy Status

Variable	Adolescents without food allergy^a^ (n = 18,582 [84.4%])		Adolescents with food allergy^a^ (n = 3,435 [15.6%])		*p*^b^
	n	(%)	n	(%)	
Sex					<.001
Female	9,288	(50.0)	2,226	(64.8)	
Male	9,294	(50.0)	1,209	(35.2)	
Age, y					.447
14	1,974	(10.6)	367	(10.7)	
15	4,489	(24.2)	866	(25.2)	
16	4,829	(26.0)	911	(26.5)	
17	4,711	(25.4)	838	(24.4)	
≥18	2,579	(13.9)	453	(13.2)	
Race/ethnicity					<.001
Hispanic/Latinos	5,358	(28.9)	858	(25.0)	
Non-Hispanic African American	1,862	(10.0)	462	(13.5)	
Non-Hispanic others	2,022	(10.9)	456	(13.3)	
Non-Hispanic White	9,340	(50.3)	1,659	(48.3)	
Alcohol use, past month	5,627	(30.3)	1,175	(34.2)	<.001
Cigarette smoking, past month	1,501	(8.1)	298	(8.7)	.240
Marijuana use, past month	3,680	(19.8)	764	(22.2)	<.001
Illicit drug use, ever^c^	2,139	(11.5)	519	(15.2)	<.001
Sleeping <6 h on an average school night	3,645	(19.6)	853	(24.8)	<.001
Bullied in school, past year	3,303	(17.8)	936	(27.3)	<.001
Bullied electronically through texting or social media, past year	2,526	(13.6)	765	(22.3)	<.001
Physically active at least 60 min/day on ≥5 days, past week	8,796	(47.3)	1,599	(46.6)	.397
Felt sad or hopeless, past year	5,863	(31.6)	1,510	(44.0)	<.001
Suicidal ideation, past year	3,186	(17.2)	866	(25.2)	<.001
Suicide attempt, past year	1,258	(6.8)	432	(12.6)	<.001
Serious suicide attempt that required medical treatment, past year^d^	341	(1.8)	160	(4.7)	<.001
Year					.001
2015	6,992	(37.6)	1,229	(35.8)	
2017	6,438	(34.7)	1,147	(33.4)	
2019	5,152	(27.7)	1,059	(30.8)	

Note: Youth Risk Behavior Survey (YRBS) databases 2015-2019.

a Respondents were asked if there are any foods that they must avoid because eating the food could cause an allergic reaction, such as skin rashes, swelling, itching, vomiting, coughing, or trouble breathing.

b The χ^2^ test was used for independence. All analyses used YRBS survey weights.

c Respondents were asked if they ever used specific illicit drugs, including cocaine, inhalants, heroin, methamphetamines, ecstasy, or hallucinogens.

d Respondents were asked if the suicide attempt resulted in an injury, poisoning, or overdose that required medical attention.

To assess the presence of an independent association between FA and serious suicide attempts necessitating medical attention, we conducted 3 progressively comprehensive multivariable logistic regression analyses. In model 1, we adjusted for demographics to discern whether demographic factors influence the association between FA and serious suicide attempts. In model 2, we expanded the analysis by incorporating sleep duration; physical activity; current use of alcohol, cigarettes, and marijuana; and lifetime use of illicit drugs to understand how the association between FA and serious suicide attempt varies with health behavior variables. In model 3, we further extended the analysis by introducing additional clinically relevant correlates of suicidal behavior, such as experiences of being bullied, feelings of sadness or hopelessness, and suicidal ideation, to ascertain whether the association between FA and suicide attempts remains independent of these covariates. Changes in adjusted odds ratios (AORs) and their 95% CIs across models were examined to evaluate variations in associations between sets of independent variables and the dependent variable. The model’s goodness of fit was assessed using the Hosmer-Lemeshow test and the log likelihood ratio test.^23^^,^^24^ The Hosmer-Lemeshow test assessed the fit of the final model to the data, whereas the log likelihood ratio test compared the fit of 3 nested models. The Hosmer-Lemeshow test statistic (χ^2^ = 7.65, *p* = .468) suggested that the final model fits the data at an acceptable level. Each model in Table 3 presents the log likelihood value, the χ^2^ associated with the change in −2 log likelihood, which measures the improvement in model fit. In all tests, a *p* value < .05 was interpreted as statistically significant.

**TABLE 3:** Multivariable Analyses Examining Associations of Food Allergy and Serious Suicide Attempt Among 22,103 High School Adolescents

Independent variables	Serious suicide attempt^a^					
	Model 1: Demographics		Model 2: Demographics + health behaviors		Model 3: Demographics + health behaviors + psychological variables + SI	
	AOR	(95% CI)	AOR	(95% CI)	AOR	(95% CI)
FA^b^	**2.28**	**(1.78-2.91)∗∗**	**2.02**	**(1.54-2.65)∗∗**	**1.63**	**(1.23-2.16)∗∗**
Sex						
Female	**2.19**	**(1.65-2.90)∗∗**	**2.39**	**(1.81-3.17)∗∗**	1.28	(0.94-1.74)
Male	Reference	(1.00)	Reference	(1.00)	Reference	(1.00)
Race/ethnicity						
Hispanic/Latinos	**1.44**	**(1.09-1.91)∗**	**1.44**	**(1.05-1.97)∗**	**1.71**	**(1.24-2.35)∗∗**
Non-Hispanic African American	1.13	(0.78-1.65)	1.46	(0.99-2.16)	**2.37**	**(1.59-3.55)∗∗**
Non-Hispanic others	**1.47**	**(1.01-2.14)∗**	**1.69**	**(1.11-2.56)∗**	**1.68**	**(1.06-2.66)∗∗**
Non-Hispanic White	Reference	(1.00)	Reference	(1.00)	Reference	(1.00)
Sexual identity						
Heterosexual	Reference	(1.00)	Reference	(1.00)	Reference	(1.00)
Sexual minority (gay, lesbian, bisexual)	**4.07**	**(3.06-5.41)∗∗**	**2.60**	**(1.92-3.52)∗∗**	1.18	(0.88-1.59)
Unsure	**1.68**	**(1.09-2.59)∗**	1.38	(0.85-2.23)	0.69	(0.41-1.17)
Alcohol use, past month	—		1.29	(0.93-1.78)	1.14	(0.85-1.53)
Cigarette smoking, past month	—		**1.91**	**(1.32-2.78)∗∗**	**1.66**	**(1.15-2.41)∗∗**
Marijuana use, past month	—		**2.33**	**(1.66-3.27)∗∗**	**1.84**	**(1.35-2.50)∗∗**
Illegal drug use, ever^c^	—		**2.83**	**(2.16-3.71)∗∗**	**1.77**	**(1.33-2.34)∗∗**
Sleeping <6 h on an average school night	—		**1.82**	**(1.46-2.26)∗∗**	1.15	(0.93-1.42)
Physically active at least 60 min/day on ≥5 days, past week	—		1.09	(0.82-1.44)	1.18	(0.87-1.59)
Bullied in school, past year	—		—		**1.40**	**(1.04-1.89)∗**
Bullied electronically through texting or social media past year	—		—		**1.83**	**(1.37-2.44)∗∗**
Felt sad or hopeless, past year	—		—		**3.97**	**(2.36-6.66)∗∗**
Suicidal ideation, past year	—		—		**40.99**	**(20.70-81.15)∗∗**
Model fit						
Log likelihood value	−2222.95		−2012.40		−1427.47	
Difference in *df*	—		5		5	
Likelihood ratio test	—		**421.1∗∗**		**1169.9∗∗**	

Note: Youth Risk Behavior Survey (YRBS) databases 2015-2019. All analyses adjust for age and survey-years and use YRBS survey weights. Boldface type indicates statistical significance at the *p* < .05 level. AOR = adjusted odds ratio; FA = food allergy; SI = suicidal ideation.

∗*p* < .05; ∗∗ *p* < .01.

a Respondents were asked if the suicide attempt resulted in an injury, poisoning, or overdose that required medical attention.

b Respondents were asked if there are any foods that they must avoid because eating the food could cause an allergic reaction, such as skin rashes, swelling, itching, vomiting, coughing, or trouble breathing.

c Respondents were asked if they ever used specific illicit drugs, including cocaine, inhalants, heroin, methamphetamines, ecstasy, or hallucinogens.

The primary analyses focused on suicide attempts necessitating medical treatment. We conducted a sensitivity analysis to evaluate the association of FA with all suicide attempts (regardless of the need for medical intervention). Further, due to demographic differences in the race variable between included and excluded participants (Table S1, available online), we conducted another sensitivity analysis. We included participants with missing alcohol intake data (n = 2,071) and reevaluated demographic disparities; no differences in demographics were found in this reevaluation (Table S2, available online). We reevaluated the association between FA and serious suicide attempts using multivariate logistic regression (Table S3, available online). Lastly, a sensitivity analysis using multinomial logistic regression (Table S4, available online) was performed to examine if the severity of suicide attempts affects the association between FA and suicide. The outcomes were categorized as no suicide attempts, suicide attempts not necessitating medical intervention, and serious suicide attempts. All statistical analyses were performed using Stata 17.0 software (StataCorp LLC, College Station, Texas), taking into account the complex sampling design. We employed svy commands along with the population weights and survey design variables provided by the YRBS to ensure the appropriate handling of the survey data.

## Results

Tables 1 and 2 present the descriptive characteristics of the 22,017 participants. FA was observed to be more prevalent among individuals who reported serious suicide attempts compared with all other respondents (31.9% vs 15.2%). Table 3 displays the outcomes of the adjusted logistic regression analyses concerning serious suicide attempts. In model 1, adjusted for demographics, respondents with FA were more likely to report serious suicide attempts (AOR 2.28, 95% CI 1.78-2.91) compared with respondents without FA.

In model 2, adjusted for demographics and health behaviors, respondents with FA were more likely to report serious suicide attempts (AOR 2.02, 95% CI 1.54-2.65) compared with respondents without FA, but the AOR was slightly diminished. Upon incorporating the remaining variables in model 3 (feelings of sadness/hopelessness, suicidal ideation, and experiences of being bullied), the association between FA and serious suicide attempts remained statistically significant (AOR 1.63, 95% CI 1.23-2.16). However, again, the AOR showed a slight decrease. Model 3 also revealed that serious suicidal behavior was more prevalent among members of racial and ethnic minority groups, respondents reporting past-month use of cigarettes or marijuana, youth who experienced bullying on the school property or electronically (via texting, Facebook, or Instagram) within the past year, and youth reporting feelings of sadness or hopelessness and suicidal ideation within the past year.

FA remained significant in the sensitivity analysis when the outcome was expanded to encompass all suicide attempts and not solely attempts requiring medical attention. The AORs of 3 models examining the association between FA and all suicide attempts using binary logistic regressions exhibited a consistent trend of diminishing AORs as the models were expanded: model 1 (AOR 1.78, 95% CI 1.54-2.06), model 2 (AOR 1.66, 95% CI 1.41-1.95), model 3 (AOR 1.35, 95% CI 1.09-1.68). The sensitivity analysis assessing FA and serious suicide attempts, including participants with missing alcohol intake, remained significant (Table S3, available online). Additionally, the sensitivity analysis using multinomial logistic regression to assess the association between FA and severity of suicide attempts remained significant for suicide attempts not requiring medical attention (AOR 1.22, *p* = .024) and for serious suicide attempts (AOR 1.68, *p* < .001) (Table S4, available online).

## Discussion

In a comprehensive study encompassing a large, nationally representative sample, we observed that adolescents with FA were more likely to report making a suicide attempt than adolescents without FA. This association persisted independently of demographics (age, sex, race, ethnicity, and sexual orientation) and clinical risk markers for suicidal behavior (such as feelings of sadness/hopelessness, alcohol consumption, cigarette smoking, marijuana usage, illicit drug use, experiences of being bullied, insufficient sleep duration, and serious suicidal ideation). Furthermore, FA demonstrated an association with both serious suicide attempts requiring medical attention and suicide attempts that did not necessitate medical intervention. Notably, the persistence of the association between FA and serious suicide attempts even after adjustment for covariates, including suicidal ideation, is significant, especially considering the prospective link between serious suicide attempts and mortality.^25^

To the best of our knowledge, this study represents the first exploration of the association between FA and serious suicide attempts among adolescents. This finding underscores the importance for clinicians treating adolescents with FA to be cognizant of the increased risk for serious for serious suicidal behavior. Maintaining clinical vigilance is crucial, both within traditional health care delivery settings and in community-based environments, including schools.^26^ Health care practitioners have a pivotal role in identifying and addressing suicide risk in adolescents with FA. By routinely screening for mental health issues in patients with FA and observing warning signs, practitioners can identify at-risk individuals.^27^ Providing appropriate interventions to individuals exhibiting suicidal ideation and ensuring access to mental health support and resources are crucial steps.^27^ Practitioners can promote mental health and well-being in this vulnerable population through a comprehensive and proactive approach.^28^^,^^29^

Our study has several limitations that should be acknowledged when interpreting the study findings. Given the cross-sectional nature of this study, causal associations between predictors and outcomes cannot be inferred. The high degree of missing data for FA and suicide attempt questions (39%) raises concerns about data completeness and could introduce bias. By incorporating multiple covariates into the analyses, many students were excluded due to missing data on alcohol use, potentially leading to selection bias. However, our sensitivity analyses including these excluded youth (Table S3, available online) yielded similar results, mitigating concerns about the impact of missing data on the robustness of our findings. Additionally, students who were absent on the day the YRBS survey was administered or had dropped out of school were not included in the survey. The data on FA, suicide attempts, feelings of sadness or hopelessness, sleep patterns, bullying experiences, and other covariates are self-reported, which introduces potential biases such as social desirability bias. The study did not assess the presence of neurodevelopmental disorders (eg, intellectual disability, autism spectrum disorder, or attention-deficit/hyperactivity disorder) as well as family dysfunction and violence, known influencers of suicidal behavior in adolescents.^30^^,^^31^ Furthermore, data on suicides, the severity of FA symptoms and associated morbidity, other allergies,^18^^,^^32^ and medications used to manage FA were unavailable.

Our study suggests several avenues for future research. First, it is essential to replicate the findings reported here in other age groups and datasets, while also considering the effects of other allergies,^18^^,^^32^^,^^33^ medications used to manage FA (such as glucocorticoids,^34^ antihistamines,^35^ and montelukast sodium^36^), experiences of family violence, and the presence of neurodevelopmental disorders, all of which are associated with suicidal behavior in adolescents. Further investigations are warranted to explore biopsychosocial pathways that underlie the influence of FA on serious suicide attempts. For instance, FA has been linked to an increase in inflammatory biomarkers such as C-reactive protein,^37^ interleukin-1β,^38^ interleukin-6,^39^ and tumor necrosis factor,^39^ all of which are also associated with suicide risk,^40^^,^^41^ suggesting a potential link between FA and suicide mediated by immune dysregulation. Moreover, to mitigate the limitation of social desirability bias in our study, future research endeavors might contemplate integrating supplementary validation measures from external sources and other studies. Lastly, interventional studies could provide insights into whether treating FA, either through behavioral approaches by minimizing exposure to allergens or pharmacologically through immunotherapy or desensitization, might mitigate suicide risk.^42^^,^^43^

Health care practitioners caring for adolescents with FA should be cognizant of their increased susceptibility to suicidal behavior. Our finding calls for future prospective studies involving diverse population groups and research to elucidate the mechanisms by which FA is associated with suicide risk.

## CRediT authorship contribution statement

**Saurabh Kalra:** Writing – review & editing, Writing – original draft, Visualization, Validation, Supervision, Software, Resources, Project administration, Methodology, Investigation, Formal analysis, Data curation, Conceptualization. **Evan M. Kleiman:** Writing – review & editing. **Shireen L. Rizvi:** Writing – review & editing. **Irina B. Grafova:** Writing – review & editing. **Paul R. Duberstein:** Writing – review & editing, Validation. **Deepak Kalra:** Writing – review & editing, Writing – original draft, Visualization, Validation, Supervision, Resources, Project administration, Methodology, Investigation, Formal analysis.
