# Supplementary material for: Increased Risk of Suicide Attempts Among Adolescents With Food Allergy in the United States
Source: JAACAP Open. 2024 Nov 29;3(3):548–56. doi: 10.1016/j.jaacop.2024.11.005 (PMC12414298; doi:10.1016/j.jaacop.2024.11.005)
Supplement: Supplemental Tables [file mmc1.docx]

**Table S1: Testing for demographic differences between study participants included and excluded in this study, Youth Risk Behavior Survey (YRBS) (2015-19), United States**

|  | **Variable** |  | Participants included  n=22,017  n(%) | Participants excluded  n=4,034  n(%) | p value^c^ |
| --- | --- | --- | --- | --- | --- |
| Sex: Female | | | 11,508 (52.3) | 2,056 (51.0) | 0.736 |
| Male | | | 10,509 (47.7) | 1,978 (49.0) |  |
| Age Group: 14 y old | | | 2,359 (10.7) | 413 (10.2) | 0.743 |
| 15 y old | | | 5,344 (24.3) | 1,087 (25.0) |  |
| 16 y old | | | 5,736 (26.1) | 1,046 (25.9) |  |
| 17 y old | | | 5,548 (25.2) | 986 (24.4) |  |
| 18 y old or older | | | 3,030 (13.8) | 582 (14.4) |  |
| Race/Ethnicity: Non-Hispanic White | | | 10,993 (49.9) | 1,745 (43.3) | 0.042 * |
| Non-Hispanic African American | | | 2,325 (10.6) | 589 (14.6) |  |
| Hispanic/Latinos | | | 6,219 (28.3) | 1,271 (31.5) |  |
| Non-Hispanic Others | | | 2,480 (11.3) | 429 (10.6) |  |
| Note: All analyses utilize YRBS survey weights. The reported p-values are based on chi-square tests for independence. Participants excluded from the analysis were those with missing data on cigarette smoking, alcohol use, marijuana use, illicit drug use, feelings of sadness or hopelessness, bullying at school property, electronic bullying through social media or texting, physical activity, sexual identity, and suicidal ideation. * p < 0.05; ** p < 0.01 | | | | | |

**Table S2: Testing for demographic differences among study participants included with missing alcohol intake data, Youth Risk Behavior Survey (YRBS) (2015-19), United States**

|  | **Variable** |  | Participants included  n=23,923  n(%) | Participants excluded  n=2,493  n(%) | p value^c^ |
| --- | --- | --- | --- | --- | --- |
| Sex: Female | | | 12,480 (52.2) | 1,232 (49.4) | 0.824 |
| Male | | | 11,443 (47.8) | 1,261 (50.6) |  |
| Age Group: 14 y old | | | 2,541 (10.6) | 290 (11.4) | 0.534 |
| 15 y old | | | 5,826 (24.4) | 650 (25.4) |  |
| 16 y old | | | 6,239 (26.1) | 653 (25.6) |  |
| 17 y old | | | 6,019 (25.2) | 598 (23.4) |  |
| 18 y old or older | | | 3,298 (13.8) | 364 (14.3) |  |
| Race/Ethnicity: Non-Hispanic White | | | 11,708 (48.9) | 1,074 (48.3) | 0.542 |
| Non-Hispanic African American | | | 2,630 (11.0) | 296 (13.3) |  |
| Hispanic/Latinos | | | 6,906 (28.9) | 614 (27.6) |  |
| Non-Hispanic Others | | | 2,679 (11.2) | 238 (10.7) |  |
| Notes: All analyses utilize YRBS survey weights. The reported p values are based on chi-square tests for independence. Participants excluded from the analysis were those with missing data on cigarette smoking, marijuana use, illicit drug use, feelings of sadness or hopelessness, bullying at school property, electronic bullying through social media or texting, physical activity, sexual identity, and suicidal ideation. | | | | | |

**Table S3: Multivariable analyses examining associations of food allergy and serious suicide attempt among 23,923 high-school adolescents (including respondents with missing values for alcohol intake survey question), Youth Risk Behavior Survey (YRBS) (2015-19), United States**

|  | Serious Suicide Attempt that resulted in an injury, poisoning, or overdose that required medical attention^a^ (AOR (95% CI)) | | |
| --- | --- | --- | --- |
| Independent variables | Model 1  Demographics | Model 2 Demographics + health behaviors | Model 3  Demographics +  health behaviors + psychological variables + SI |
| Food Allergy (FA)^a^ | **2.39 (1.88-3.04)**** | **2.14 (1.65-2.77)**** | **1.63 (1.23-2.17)**** |
| Sex: Female | **2.43 (1.89-3.14)**** | **2.64 (2.04-3.42)**** | 1.21 (0.90-1.63) |
| Male | Ref. (1.00) | Ref. (1.00) | Ref. (1.00) |
| Race/Ethnicity: Non-Hispanic White | Ref. (1.00) | Ref. (1.00) | Ref. (1.00) |
| Non-Hispanic African American | 1.22 (0.87-1.70) | **1.41 (1.05-1.99)*** | **2.15 (1.49-3.10)**** |
| Hispanic/Latinos | **1.48 (1.11-1.97)**** | **1.47 (1.08-1.99)**** | **1.72 (1.28-2.32)**** |
| Non-Hispanic Others | **1.52 (1.04-2.20)*** | **1.65 (1.11-2.46)*** | 1.57 (0.99-2.47) |
| Cigarette smoking, past month | - | **2.49 (1.77-3.49)**** | **1.90 (1.33-2.71)**** |
| Marijuana use, past month | - | **2.41 (1.72-3.40)**** | **1.79 (1.31-2.45)**** |
| Illegal drug use, ever ^b^ | - | **3.10 (2.40-4.00)**** | **1.80 (1.37-2.35)**** |
| Sleeping less than 6 hours on an average school night | - | **2.04 (1.66-2.51)**** | 1.19 (0.98-1.46) |
| Physically active at least 60 minutes per day on 5 or more days, past week |  | 0.96 (0.75-1.24) | 1.09 (0.83-1.42) |
| Bullied in school, past year | - | - | **1.40 (1.05-1.86)*** |
| Bullied electronically through texting, or social media, past year | - | - | **1.84 (1.39-2.44)**** |
| Felt sad or hopeless, past year | - | - | **3.30 (2.14-5.11)**** |
| Suicidal ideation, past year | - | - | **40.0 (21.92-72.96)**** |
| Model fit:  Log Likelihood value | -2846.23 | -2485.29 | -1774.61 |
| Difference in *df* |  | 5 | 4 |
| Likelihood-ratio test |  | 721.9 ** | 1421.4 ** |
| Note: All analyses adjust for age and survey-years and utilize YRBS survey weights. Abbreviations: AOR = adjusted Odds Ratio; CI = Confidence Interval; * p < 0.05; ** p < 0.01. Bold values indicate statistical significance at the p < 0.05 level.  ^a^ Respondents were asked if there are any foods that they must avoid because eating the food could cause an allergic reaction, like skin rashes, swelling, itching, vomiting, coughing, or trouble breathing.  ^b^ Respondents were asked if they ever used specific illicit drugs, including cocaine, inhalants, heroin, methamphetamines, ecstasy, or hallucinogens. | | | |

**Table S4: Multinomial logistic regression sensitivity analysis to assess the relative risk between food allergy and different categories of suicide attempts, Youth Risk Behavior Survey (YRBS) (2015-19), United States**

| Suicide attempts | Independent variable | Adjusted odds ratio | St.Err. | t-value | p value | [95% Conf | Interval] | Sig |
| --- | --- | --- | --- | --- | --- | --- | --- | --- |
| None | (base outcome) |  |  |  |  |  |  |  |
| Suicide attempts not requiring medical attention | Food Allergy | 1.22 | .11 | 2.26 | .024 | 1.03 | 1.45 | * |
| Suicide attempts requiring medical attention | Food Allergy | 1.68 | .20 | 4.42 | <0.001 | 1.34 | 2.12 | ** |
| Note: * p < 0.05; ** p < 0.01. Analysis is adjusted for survey years, sex, age, race, sexual identity, within past-month use of alcohol, cigarette smoking, and marijuana, physical activity (<60 minutes for 5 days in the last week), illicit drug use, feelings of sadness or hopelessness, bullying victimization, <6 hours of sleep, and suicidal ideation. | | | | | | | | |
